# Prediabetes Phenotype Influences Improvements in Glucose Homeostasis with Resistance Training

**DOI:** 10.1371/journal.pone.0148009

**Published:** 2016-02-03

**Authors:** Joshua D. Eikenberg, Jyoti Savla, Elaina L. Marinik, Kevin P. Davy, John Pownall, Mary E. Baugh, Kyle D. Flack, Soheir Boshra, Richard A. Winett, Brenda M. Davy

**Affiliations:** 1 Department of Internal Medicine, Carilion Clinic—Virginia Tech Carilion School of Medicine, Roanoke, Virginia, United States of America; 2 Center for Gerontology & Department of Human Development, Virginia Tech, Blacksburg, Virginia, United States of America; 3 Department of Human Nutrition, Foods, and Exercise, Virginia Tech, Blacksburg, Virginia, United States of America; 4 Department of Psychology, Virginia Tech, Blacksburg, Virginia, United States of America; Weill Cornell Medical College Qatar, QATAR

## Abstract

**Purpose:**

To determine if prediabetes phenotype influences improvements in glucose homeostasis with resistance training (RT).

**Methods:**

Older, overweight individuals with prediabetes (n = 159; aged 60±5 yrs; BMI 33±4 kg/m^2^) completed a supervised RT program twice per week for 12 weeks. Body weight and composition, strength, fasting plasma glucose, 2-hr oral glucose tolerance, and Matsuda-Defronza estimated insulin sensitivity index (ISI) were assessed before and after the intervention. Participants were categorized according to their baseline prediabetes phenotype as impaired fasting glucose only (IFG) (n = 73), impaired glucose tolerance only (IGT) (n = 21), or combined IFG and IGT (IFG/IGT) (n = 65).

**Results:**

Chest press and leg press strength increased 27% and 18%, respectively, following the 12-week RT program (both *p*<0.05). Waist circumference (-1.0%; pre 109.3±10.3 cm, post 108.2±10.6 cm) and body fat (-0.6%; pre 43.7±6.8%, post 43.1±6.8%) declined, and lean body mass (+1.3%; pre 52.0±10.4 kg, post 52.7±10.7 kg) increased following the intervention. Fasting glucose concentrations did not change (*p*>0.05) following the intervention. However, 2-hr oral glucose tolerance improved in those with IGT (pre 8.94±0.72 mmol/l, post 7.83±1.11 mmol/l, *p*<0.05) and IFG/IGT (pre 9.66±1.11mmol/l, post 8.60±2.00 mmol/l) but not in those with IFG (pre 6.27±1.28mmol/l, post 6.33± 1.55 mmol/l). There were no significant changes in ISI or glucose area under the curve following the RT program.

**Conclusions:**

RT without dietary intervention improves 2-hr oral glucose tolerance in individuals with prediabetes. However, the improvements in glucose homeostasis with RT appear limited to those with IGT or combined IFG and IGT.

**Trial Registration:**

ClinicalTrials.gov: NCT01112709

## Introduction

Approximately 14.3% of Americans are estimated to suffer from type 2 diabetes mellitus (T2D) and 38.0% are estimated to suffer from prediabetes [1]. Individuals with prediabetes are at high risk with up to 70% eventually progressing to T2D over their lifetimes [2]. However, not all individuals with prediabetes progress at the same rate. The annual incidence of T2D in individuals with IGT, IFG, or combined IFG/IGT is 6.1%, 7.0%, and 14.0%, respectively [3].

The pathophysiology of IGT differs from that of IFG. Impaired fasting glucose and IGT are associated with impaired glucose-stimulated insulin secretion, however, the pattern of β-cell dysfunction in each case is distinct [4]. Impaired fasting glucose is associated with impaired early phase insulin secretion but supranormal second-phase insulin secretion resulting in a normal oral glucose tolerance test [5]. Impaired glucose tolerance is associated with both impaired early and late phase insulin secretion [5]. Insulin sensitivity also differs in IGT and IFG. Impaired glucose tolerance is associated with reduced insulin-stimulated whole-body glucose disposal [4] compared with normal glucose tolerance. In contrast, IFG is associated with normal or near-normal insulin-stimulated whole-body glucose disposal [4,6]. Because 90% of glucose disposal occurs in skeletal muscle, these findings suggest that the skeletal muscle is insulin resistant in IGT, but normal or near normal in IFG [4]. Furthermore, IFG and IGT are associated with hepatic insulin resistance. However, IFG is associated with more severe hepatic insulin resistance than IGT [7].

The efficacy of lifestyle interventions to improve glycemia may differ according to prediabetes phenotype given the differences in their pathophysiology. Interventions aimed at modifying both diet and physical activity in individuals with prediabetes have demonstrated efficacy in improving 2-hr oral glucose tolerance [8–10] and FPG [8,11], while other studies have failed to demonstrate improvements in 2-hr oral glucose tolerance [11–13] and FPG [10,12,13]. Some evidence suggests that dietary patterns may affect 2-hr oral glucose tolerance but not FPG [14] suggesting that IGT may be more responsive to dietary interventions than IFG. Taken together, these observations suggest that a “personalized medicine” approach to diabetes prevention could allow clinicians to tailor lifestyle recommendations to their patients’ needs [15].

Resistance training (RT) improves 2-hr oral glucose tolerance [16], FPG [17,18], insulin sensitivity [17], and hemoglobin A1C in individuals with T2D [16,17,19,20], and the American Diabetes Association recommends that individuals with T2D engage in RT [21]. Resistance training may also reduce an individual’s risk for developing T2D [22]. Resistance training appears to improve 2-hr oral glucose tolerance in healthy older adults [23] and in individuals with prediabetes [16]. However, there is conflicting evidence. Resistance training did not improve insulin-stimulated glucose disposal or reduce endogenous glucose production in healthy or prediabetic postmenopausal women [24] or improve FPG in prediabetic individuals [25]. In addition, these [24,25] and other [16] studies are often limited by small sample sizes, and to date, no studies have investigated whether prediabetes phenotype impacts the efficacy of RT as a diabetes risk-reduction approach. Therefore, the purpose of this investigation was to determine the influence of prediabetes phenotype (IFG, IGT, and combined IFG/IGT) on improvements in glucose homeostasis with RT.

## Materials and Methods

### Participants

**Fig 1:**
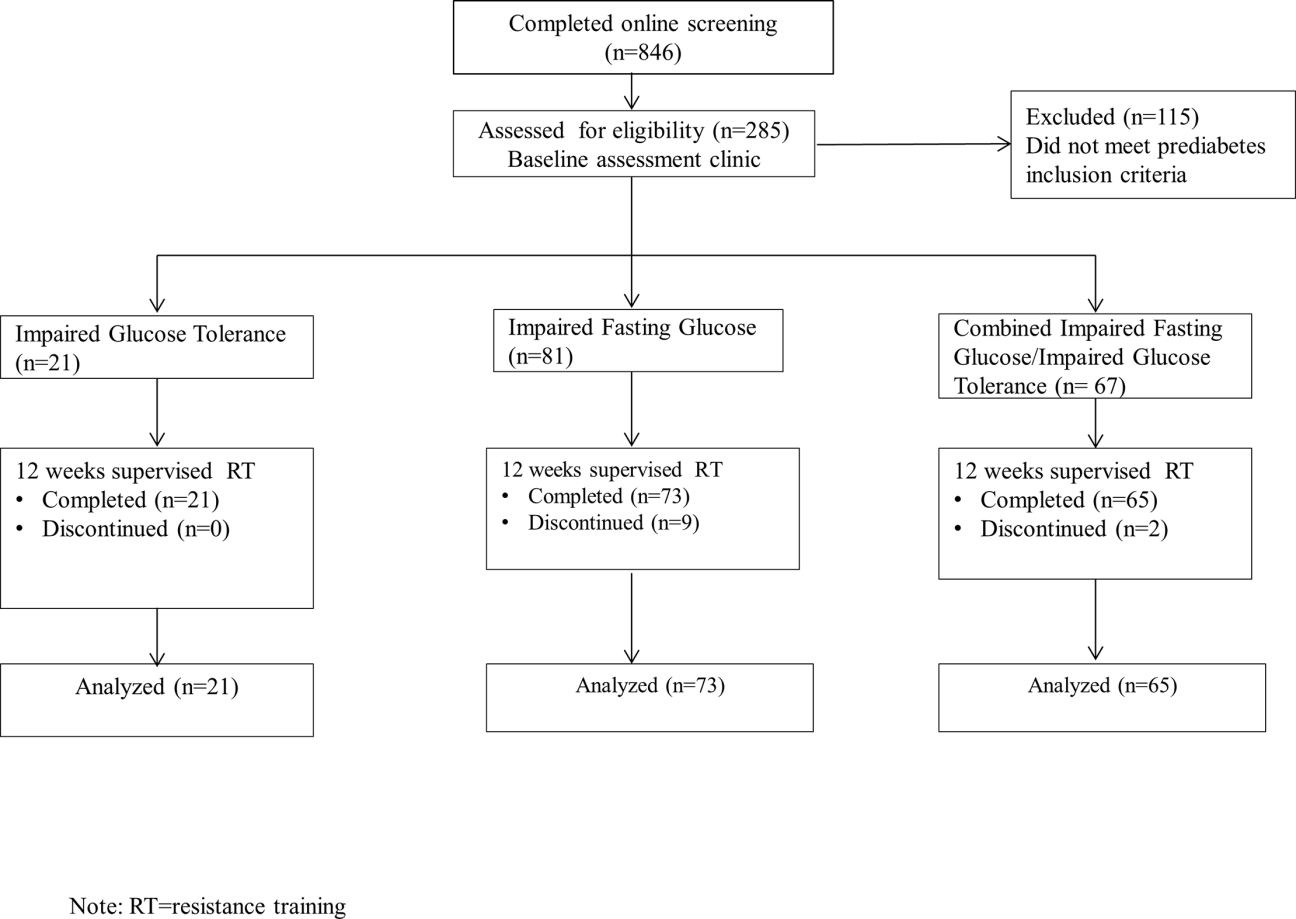
Flow diagram.

Men and women aged 50–69 years were recruited by public advertisement from January 2011 to September 2012 and were initially screened through an online process. In order to participate, individuals were required to be weight stable (±2 kg in the past year) with a BMI of 25–39.9 kg/m^2^, and sedentary or minimally physically active (<150 min/wk of moderate or <60 min/wk of vigorous physical activity) and had not engaged in RT for at least one year prior to enrollment. Individuals were excluded if they were current smokers, had been diagnosed with cardiovascular disease, diabetes, pulmonary, liver or kidney disease, or had any musculoskeletal disabilities. Individuals meeting eligibility criteria were then screened for prediabetes, and invited to enroll in the study if they met the Diabetes Prevention Program inclusion criteria for FPG or 2-hr oral glucose tolerance [26]: a FPG of 95–125 mg/dL (5.3–6.9 mmol/l) and/or a plasma glucose of 140–199 mg/dL (7.8–11.0 mmol/l) 2 h after an oral load of 75g dextrose. Participants obtained medical clearance for participation from their primary care providers. The flow diagram of this study is shown in Fig 1. This investigation utilized the results of the initial 12-week supervised training phase of a 15-month faded-contact intervention trial, the Resist Diabetes trial [27]. The study was approved by the Institutional Review Board at Virginia Tech. The nature, purpose, risks, and benefits of the study were explained before obtaining written informed consent.

### Resistance Training Protocol

The training program consisted of 12 weeks of progressive RT twice per week, supervised by an American College of Sports Medicine (ACSM)-Certified Personal Trainer®, and delivered individually to participants in a clinical research facility. Exercises were performed using resistance machines (NitroPlus, Nautilus, Inc., Vancouver, WA, USA) which included leg press, leg extension, seated leg curl, calf raise, lower back, chest press, pulldown, row, shoulder press, seated dip, rotary torso, and abdominal crunch movements. One set of 6–12 repetitions was performed, with the initial resistance selected by the trainer using a weight judged by the participant as “hard” on a standard (6–20) perceived exertion scale. Each repetition was performed throughout the entire range of motion and was comprised of a three-second concentric phase and a three-second eccentric phase. The resistance was increased by 5% for each exercise after a participant was able to complete eight repetitions for two consecutive or two out of three RT sessions. Adherence to the twice weekly training sessions was monitored by the study coordinator (ELM). Individuals were instructed to maintain their habitual dietary intake and physical activity patterns.

### Oral Glucose Tolerance Test

An oral glucose tolerance test was administered (75 g Fisherbrand, Fisher Healthcare, Houston, TX, USA) after a 12-h fast with plasma glucose sampling at 0, 10, 20, 30, 60, 90 and 120 min before and after the completion of the 12-week RT program. Plasma glucose was determined by a YSI 2700 Select Biochemistry Analyzer (YSI, Inc., Yellow Springs, OH). Two- hour oral glucose tolerance was the plasma glucose at 120 minutes following the oral glucose load. Serum insulin was analyzed by commercially available enzyme linked immunoassay (ALPCO, Salem, NH; Insulin IMMULITE Kit, Siemens, Erlangen, Germany). Estimated insulin sensitivity index (ISI) was calculated as described by Matsuda and Defronza [28]:

$$\text{ISI}=\frac{10,000}{\sqrt{\text{FPG x FPI x}\;(\text{mean OGTT glucose concentration x mean OGTT insulin concentration})}}$$

FPG: fasting plasma glucose; FPI: fasting plasma insulin; OGTT: 2-hr oral glucose tolerance test

### Anthropometrics, Body Composition, and Blood Pressure

Height and weight were determined using a wall-mounted stadiometer and a digital scale (Healthometer ProPlus, Pelstar, Brideview, IL, USA), respectively. Waist circumference was measured using a Gulick tape measure (Gulick, Country Technology, Gays Mill, WI) at the level of the umbilicus. Body composition (fat mass, lean body mass) was assessed using dual-energy x-ray absorptiometry (Prodigy Advance, software version 11.40.004, GE Healthcare Lunar, Madison, WI). Body mass index (BMI) was calculated as weight(kg)/height(m)^2^. Blood pressure was measured in the seated position using an automated Dinamap XL vital signs monitor (model 9300, J & J Medical, Tampa, FL) [29].

### Strength Assessment

Strength was assessed at baseline and at 12 weeks by performing a three repetition maximum (3RM) test on the chest press and leg press, according to ACSM guidelines [30]. Prior to baseline assessments, all participants had an extended orientation to and practice with the movements. Following orientation, the procedures consisted of a brief warm-up on a treadmill or stationary bike, a specific warm-up on each RT machine, and then the performance of five controlled repetitions where the last repetition was judged as ‘hard’ on a relative perceived exertion scale. Participants then rested for five minutes. The resistance was then increased 10–20% on each machine beyond the resistance used for completing five repetitions. Participants performed three controlled repetitions at a relative perceived exertion during the last repetition of ‘very hard’ with an inability to perform a fourth repetition. If this level of perceived exertion was not reached, the participant rested for three minutes, the resistance was increased 5–10%, and the procedure was repeated.

### Habitual Physical Activity Assessment

Non-RT physical activity was assessed online using the Aerobics Center Longitudinal Study Questionnaire [31] at baseline and at 12 weeks. Total physical activity minutes and MET-hr per week were calculated.

### Dietary Assessment

Three 24-hour dietary recalls were obtained for each participant prior to and following completion of the 12-week RT program by a trained research dietary technician. The first recall was done in-person and the second and third were obtained via phone. Recalls were completed within a two-week timeframe at each of the two assessment points. Diagrams of food models were used to assist participants in accurate estimation of portion sizes. The average of the three recalls was used to calculate energy and macronutrient intake using Nutrition Data Systems for Research 2010 software (Nutrition Coordinating Center, University of Minnesota).

### Statistical Analysis

The sample size calculation was based on the sample size calculation for the Resist Diabetes clinical trial. The study design, including the method of sample size calculation, is detailed by Marinik et al. [27]. The Resist Diabetes study was a 15-month randomized controlled trial including men and women aged 50–69 years (n = 170) with prediabetes. All participants first followed the same standard, supervised 3-month Initiation phase with RT. After the 3-month Initiation phase, participants (n = 159) were randomly assigned to one of two maintenance conditions for 6 months: 1. a long-term, higher fidelity, social cognitive theory (SCT) intervention using interactive, self-regulation procedures with tailored web-based and faded personal contact, or 2. A usual care condition with SCT content but with generic web-based and more minimal contact. For both conditions, contact ended after 6 months, but participants were expected to continue RT at their respective facilities. Assessments were completed at baseline, at the end of the common Initiation phase (3 months), at the end of the different maintenance intervention phase (9 months), and six months after all contact had ended (15 months from baseline), providing an assessment of maintenance with differential faded contact and with no further contact. The Resist Diabetes trial was designed to detect differences between these two groups in regards to fasting plasma glucose, 2-hr oral glucose tolerance, and increases in strength. Using a Monte Carlo sample size estimation approach in MPlus [32], we estimated that N = 55 per group would provide sufficient power to detect significant group differences in change over the four assessment points (baseline, 3 months, 9 months and 15 months) for achievement of normal fasting plasma glucose, 2-hr plasma glucose concentration, and increases in strength (α = .05, effect size = 0.60). The effect size was informed by data from prior studies that do not have a component of RT but have employed similar measures as in the Resist Diabetes trial [33], as well as from prior studies with RT and T2D [19,20,34–39] that have shown standard deviation estimates of 15% with a maximum difference between placebo and intervention groups to be approximately 15–20% at the final time point, comparable to a medium effect size. For the hypothesis that positive changes in glucose homeostasis and strength will be mediated by self-efficacy, self-regulation, adherence, and outcome expectancies, we estimated a sample size of 55 per group would provide sufficient power (>.80) to detect a significant medium to large mediation effect size using a regression framework. Testing the mediation model in a structural equation modeling framework [40,41] we estimated that a sample of 110 participants could detect a close fitting mediation model. A sample of 170 participants was enrolled to ensure that 110 participants complete all four assessment points of the study, allowing for 35% attrition rate.

Results are reported as means ± standard deviation. One-way ANOVAs were used to evaluate differences in subject characteristics and other dependent variables between the three prediabetes groups at baseline. Repeated measures ANOVAs were used to assess main and interaction effects. Tukey’s post-hoc test was used for post-hoc analyses when a significant F test was observed. Pearson’s correlation coefficient r was used to explore relationships among variables of interest.

A mixed effects linear regression model with an independent covariance structure was used to determine if group differences in glycemic variables remained after controlling for covariates. Analysis for interaction effects between the three prediabetes phenotypes was conducted on glycemic variables with significant group differences. Analysis for regression to the mean was also conducted on glycemic variables with significant group differences based on the formula for regression to the mean effect [42]. Plasma glucose area under the curve (AUC) was calculated using the trapezoidal model [43]. The SPSS Statistical package (version 22, IBM, Armonk, NY) and STATA (version 12, StataCorp LP, College Station, TX) were used to perform the statistical analyses.

## Results

### Participant Characteristics

Of the 285 participants screened for prediabetes, 170 individuals met eligibility criteria and were enrolled in the RT program. A total of 159 participants (72% female; 94% white; aged 59.6 ± 5.4 yrs) completed the 12-week RT program (i.e., 94% retention). At baseline, 73 participants (71% female) were classified as IFG, 21 participants (71% female) were classified as IGT, and 65 participants (73% female) were classified as combined IFG/IGT.

### Compliance and Adverse Events

Adherence to the RT protocol (sessions completed) was 87 ± 7%, with no difference across groups. No adverse events were reported during the 12-week supervised training phase.

### Strength, Anthropometrics, Body Composition, and Blood Pressure

Strength, anthropometric variables, and body composition at baseline and following the 12-week intervention are presented in Table 1. Chest press strength and leg press strength were not significantly different among the three groups at baseline. Chest press and leg press strength increased (both *p* < 0.05) by 27% and 18%, respectively, but there were no significant differences in strength gains between groups (Table 1). Body mass index differed among groups at baseline (*p*<0.05) with the highest level observed in the IFG group and lowest level in IFG/IGT group. However, there was no significant change in BMI following the 12-week RT program (Table 1). There was a significant reduction in waist circumference following the intervention, but the reductions were similar (*p*>0.05) between groups (Table 1). Body fat percentage decreased by ~1.4% and lean mass increased by ~1.3% (both *p*<0.05) with the intervention (Table 1). These changes appeared to be limited to those with IFG and IFG/IGT with no obvious change in those with IGT (*p*>0.05). Systolic and diastolic blood pressure declined with the RT program, but there were no differences in the reductions between the groups (data not shown).

**Table 1:** Strength, anthropometric variables, and body composition at baseline and after a 12-week resistance training program in the three different prediabetes phenotypes.

	All Participants (n = 159)		IFG (n = 73)	IGT (n = 21)	IFG/IGT (n = 65)	Group x time interaction
	Mean±SD	*p* Value^*^	Mean ± SD	Mean ± SD	Mean ± SD	*p* Value
Chest press strength (kg)						
Baseline	33.7±11.7		33.1 ± 12.3	34.7 ± 10.2	34.2 ± 11.5	
12 weeks	42.8±14.8	<0.001	42.0 ± 15.5	44.5 ± 12.7	43.2 ± 14.9	0.876
Leg press strength (kg)						
Baseline	141.1±36.2		140.1 ± 37.7	142.5 ± 39.0	141.8 ± 34.0	
12 weeks	166.4±39.3	<0.001	162.8 ± 39.3	170.2 ± 42.4	169.1 ± 38.5	0.258
BMI (kg/m^2^)						
Baseline^†^	33.0 ± 3.8		32.2 ± 3.3	33.2 ± 3.7	33.8 ± 4.1	
12 weeks	33.0 ± 3.9	0.603	32.2 ± 3.4	32.9 ± 3.8	33.8 ± 4.2	0.217
WC (cm)						
Baseline	109 ± 10		108 ± 10	110 ± 9	111 ± 11	
12 weeks	108 ± 11	0.001	107 ± 10	108 ± 10	109 ± 12	0.483
Body fat %						
Baseline	43.7 ± 6.8		44.2 ± 6.9	43.0 ± 6.4	43.5 ± 6.9	
12 weeks	43.1 ± 6.8	<0.001	43.3 ± 6.9	43.0 ± 6.3	43.0 ± 7.0	0.026^‡^
Lean mass (kg)						
Baseline	52.0 ± 10.4		51.5 ± 11.2	51.2 ± 8.2	53.5 ± 10.2	
12 weeks	52.7 ± 10.7	0.001	52.3 ± 11.4	50.5 ± 8.6	53.9 ± 10.5	0.001^‡^

Abbreviations: IFG. impaired fasting glucose; IGT, impaired glucose tolerance; BMI, body mass index; WC, waist circumference.

*Time effect.

†Significant baseline difference across groups (*p* = 0.036).

‡Tukey’s post-hoc analyses did not reveal significant differences between pairs of prediabetes subtypes.

### Habitual Physical Activity and Dietary Intake

Habitual physical activity and dietary intake at baseline and following the 12-week RT program were available for 101 and 159 individuals, respectively. There were no differences (all *p*<0.05) in habitual physical activity or dietary intake between the three groups at baseline. Total energy and carbohydrate intake declined by ~2 and ~4% (both *p*<0.05), respectively, following the intervention. However, there were no differences in total energy or carbohydrate between the groups following the RT program. There were no significant changes in reported intake of other macronutrients or physical activity with the intervention (Table 2).

**Table 2:** Habitual physical activity and dietary intake at baseline and after a 12-week resistance training program in the three different prediabetes phenotypes.

	All Participants (n = 159)		IFG(n = 73)	IGT(n = 21)	IFG/ IGT (n = 65)	Group x time interaction
	Mean±SD	*p* Value^*^	Mean ± SD	Mean ± SD	Mean ± SD	*p* Value
Physical activity (min/wk)						
Baseline	132 ± 133		122 ± 73	148 ± 168	138 ± 171	
12 weeks	148 ± 140	0.118	149 ± 133	222 ± 242	124 ± 93	0.318
Physical activity (MET-hr/wk)						
Baseline	8.5 ± 8.4		7.9 ± 5.0	9.9 ± 12.8	8.7 ± 10.0	
12 weeks	9.6 ± 9.4	0.139	9.8 ± 8.8	14.3 ± 17.3	7.9 ± 5.8	0.358
Energy intake (kcal/day)						
Baseline	1807 ± 507		1744 ± 541	1944± 567	1832 ± 439	
12 weeks	1743± 462	0.034	1681 ± 454	1894 ± 498	1765 ± 453	0.978
Fat intake (g/day)						
Baseline	77 ± 30		72 ± 29	85 ± 37	79 ± 27	
12 weeks	74 ± 24	0.125	70 ± 24	78 ± 26	75 ± 24	0.711
Protein intake (g/day)						
Baseline	79 ± 23		76 ± 23	80 ± 25	81 ± 23	
12 weeks	78 ± 21	0.589	78 ± 22	76 ± 19	77 ± 21	0.142
Carbohy-drate intake (g/day)						
Baseline	201 ± 60		194 ± 67	217 ± 53	202 ± 54	
12 weeks	192 ± 62	0.018	181 ± 62	220 ± 67	195 ± 57	0.326

Abbreviations: IFG. impaired fasting glucose; IGT, impaired glucose tolerance; WC, waist circumference.

*Time effect.

### Glucose Homeostasis

**Fig 2:**
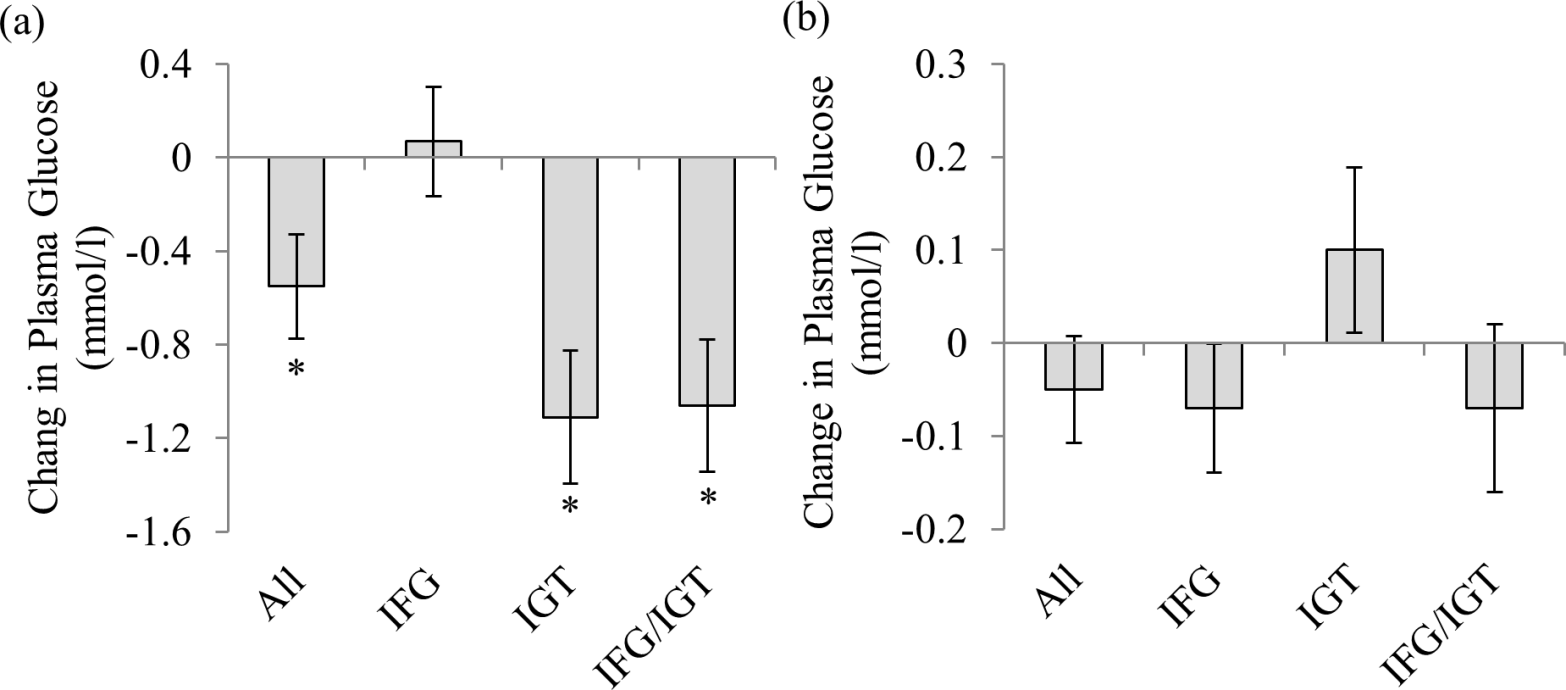
(a) Changes in 2-hour oral glucose tolerance from baseline after 12-weeks of resistance training in the pooled sample (All), participants with impaired fasting glucose (IFG), participants with impaired glucose tolerance (IGT), and participants with IFG/IGT. * *p*<0.001 vs. baseline. (b) Change in fasting glucose from baseline after 12-weeks of resistance training for the pooled sample. Error bars show standard error.

As expected, fasting glucose concentrations were higher (both *p*>0.05) at baseline in those with IFG and IFG/IGT compared with those with IGT (Table 3 and Fig 2). However, there were no significant changes in fasting glucose concentrations with the RT program. Two-hour oral glucose tolerance, i.e. plasma glucose at 120 minutes following the oral glucose load, was lower in those with IGT and IFG/IGT at baseline compared to those with IFG (both *p*<0.05). Two- hour oral glucose tolerance increased ~7% (*p*<0.05) with the 12-week RT intervention (Table 3). However, the improvements in 2-hr oral glucose tolerance with RT were evident in those with IGT (*p*<0.05) and IFG/IGT (*p* = 0.051) compared with those with IFG (Fig 2).

**Table 3:** Fasting glucose, 2-hour oral glucose tolerance, estimated insulin sensitivity index, and plasma glucose area under the curve at baseline and after a 12-week resistance training program in the three prediabetes phenotypes.

	All Participants		IFG	IGT	IFG/IGT	Group x time interaction
	Mean ± SD	*p* Value^†^	Mean ± SD	Mean ± SD	Mean ± SD	*p* Value
Fasting glucose (mmol/l)^*^						
Baseline	5.64 ± 0.47		5.63 ± 0.29	4.98 ± 0.18	5.87 ± 0.48	
12 weeks	5.59 ± 0.55	0.196	5.56 ± 0.51	5.09 ± 0.37	5.80 ± 0.54	0.274
2-hr oral glucose tolerance (mmol/l)^*^						
Baseline	8.02 ± 1.99		6.29 ± 1.29	8.93 ± 0.69	9.67 ± 1.13	
12 weeks	7.47 ± 1.99	<0.001	6.35 ± 1.53	7.83 ± 1.10	8.61 ± 1.98	<0.001^‡^
ISI^*^						
Baseline	0.507±0.775		0.077±1.069	0.476±0.601	0.254±0.149	
12 weeks	0.443±0.619	0.459	0.610±0.860	0.374±0.302	0.300±0.289	0.513
PG AUC (mmol·120 min)^*^						
Baseline	19066±2959		17178±2618	18906±1607	21043±2245	
12 weeks	18481±3167	0.031	16817±3046	18263±1852	20245±2640	0.677

Abbreviations: IFG. impaired fasting glucose; IGT, impaired glucose tolerance; ISI, insulin sensitivity index; PG, plasma glucose; AUC, area under the curve

*Sample sizes: Fasting glucose and 2 hr oral glucose tolerance (n = 159), ISI (n = 113), Plasma Glucose AUC (n = 104).

†Time effect.

‡Tukey’s post-hoc test showed significant differences between the IFG group and the IGT group and between the IFG group and the IFG/IGT group (*p*<0.001). There were no significant differences between the IGT group and the IFG/IGT group.

We conducted sensitivity analyses with and without the IFG group because baseline 2-hr oral glucose tolerance was lower among those with IFG. The sensitivity analyses indicated that 2-hr glucose concentrations decreased in the IGT (t = 5.010; *p*<0.001) and COM group (t = 4.877; *p* <0.001) following the RT program. Regression to the mean only accounted for 36%, 39%, and 31% of the change in 2-hr oral glucose tolerance in the IFG, IGT, and COM groups, respectively. The change in lean body mass was correlated with the change in 2-hr oral glucose tolerance (r = 0.172; *p* = 0.031). The significant between-group differences in 2-hr oral glucose tolerance persisted (*p* < 0.001) after adjusting for the between-group differences in lean body mass. There was no correlation between the change in body fat percentage and the change in 2-hr oral glucose tolerance.

There was no difference (*p*>0.05) in ISI between the groups at baseline. In addition, ISI did not change (*p*>0.05) following the RT program (Table 3). Plasma glucose AUC was similar (*p*>0.05) among the groups at baseline. In addition, there was a significant decrease in plasma glucose AUC with the RT program. However, there were no differences in changes in plasma glucose AUC with RT among the three groups differences (Table 3). Twenty two percent of the pooled sample, 38% of the IGT group, 27% of the IFG group, and 11% of the IFG/IGT group had glycemic values below the prediabetes range used for inclusion in the trial following the RT program. None of the participants progressed to T2D.

## Discussion

The major new finding of the present study is that the improvements in 2-hr oral glucose tolerance with RT in middle-aged and older individuals with prediabetes are limited to those with IGT or IFG/IGT. Importantly, the improvements in 2-hr oral glucose tolerance in this study occurred without any dietary intervention or any changes in non-RT physical activity. However, RT did not improve FPG in the present study. Nevertheless, individuals with prediabetes may benefit from RT in other ways. For example, RT was associated with increased strength, favorable change in body composition, and reductions in both systolic and diastolic blood pressure in this study. As such, RT appears to be an efficacious lifestyle strategy for improving a multitude of health outcomes among individuals with prediabetes.

Severely obese (BMI≥ 35), middle-aged (<60 years old) individuals with IFG/IGT appear to benefit more from metformin therapy compared with other prediabetic subgroups [2]. This observation led to the recommendation that, in addition to FPG, 2-hr oral glucose tolerance should be assessed if metformin therapy is being considered [2]. Similarly, our findings suggest that assessment of 2-hr oral glucose tolerance may potentially be used to identify individuals with prediabetes who may benefit the most from including RT as part of lifestyle modification.

Our observation that RT improves 2-hr oral glucose tolerance in individuals IGT is consistent with a previous study [16]. That RT did not improve FPG in individuals with IFG in the present study is consistent with some [19,20,25] but not all previous studies [17,18] in individuals with T2D. The reason for this discrepancy is unclear, but may be attributable to age, gender, FPG, or physical activity levels of the participants or the dose of RT. Future studies will be necessary to clarify this issue.

The mechanism by which RT may improve 2-hr oral glucose tolerance and not FPG is unclear. Impaired glucose tolerance is associated with reduced insulin-stimulated glucose disposal [44–46] and some evidence suggests RT improves insulin sensitivity in middle-age and older adults with T2D [17]. In addition, RT also improves insulin signaling at the level of skeletal muscle [47]. Taken together, our findings suggest that improvements 2-hr oral glucose tolerance and increases in lean body mass in the present study may reflect an enhancement in insulin-stimulated glucose disposal at the level of skeletal muscle. That increases in lean body mass and improvements in 2-hr oral glucose tolerance were correlated in the present study is supportive of this postulate. Future studies will be necessary to address the mechanism responsible for the prediabetes phenotypic-specific improvements in 2-hr oral glucose tolerance with RT.

The major strengths of the present study include the relatively large sample size compared with previous studies involving individuals with prediabetes and the novel comparison of the effects of RT according to prediabetes phenotype. However, there are some limitations that should also be considered. We did not include control groups who did not receive the RT intervention. Furthermore, we did not measure hemoglobin A1C in the present study and, thus, may have missed an opportunity to identify another prediabetes phenotype. We also used an indirect measure of insulin sensitivity that may not have been sensitive enough to detect small changes with the RT program. Finally, we did not measure visceral fat with computed tomography or magnetic resonance imaging in the present study. As such, we were unable to determine if there were prediabetes phenotype dependent changes in visceral fat with the RT intervention.

In conclusion, a 12-week twice weekly, full-body RT program may improve 2-hr oral glucose tolerance in middle-aged and older prediabetic individuals with IGT or combined IFG/IGT. These improvements in 2-hr oral glucose tolerance in this study occurred in the absence of any dietary intervention or change in non-RT physical activity. In contrast, FPG did not decline with the RT intervention. However, RT increased strength, favorably modified body composition, and reduced systolic and diastolic blood pressure in prediabetic middle-aged and older adults in the present study. Future studies are needed to address whether RT can be sustained beyond a 12-week supervised period, particularly when unsupervised in a community setting, and whether the benefits of RT are maintained over time. Our findings suggest that it may be possible to personalize RT recommendations based on an individual’s prediabetic phenotype.

## Supporting Information

S1 TREND Checklist TREND Checklist. (PDF)

S1 Protocol The Resist Diabetes Study Protocol. (PDF)
