# Supplementary material for: Prediabetes Phenotype Influences Improvements in Glucose Homeostasis with Resistance Training
Source: PLoS One. 2016 Feb 3;11(2):e0148009. doi: 10.1371/journal.pone.0148009 (PMC4739703; doi:10.1371/journal.pone.0148009)
Supplement: S1 Protocol — (PDF) [file pone.0148009.s002.pdf]

Once complete, upload this form as a Word document to the IRB Protocol Management System: https://secure.research.vt.edu/irb

Section 1: General Information

1.1 DO ANY OF THE INVESTIGATORS OF THIS PROJECT HAVE A REPORTABLE CONFLICT OF INTEREST? (http://www.irb.vt.edu/pages/researchers.htm#conflict)

No
Yes, explain: **This conflict has been fully disclosed and was approved for management on 2/28/09 by the associate provost, Dr. Patricia Hyer.**

1.2 WILL THIS RESEARCH INVOLVE COLLABORATION WITH ANOTHER INSTITUTION?

No, go to question 1.3
Yes, answer questions within table

IF YES
**Provide the name of the institution** [for institutions located overseas, please also provide name of country]**:**<br>**Brown University**
**Indicate the status of this research project with the other institution's IRB:**<br>Pending approval<br>Approved<br>Other institution does not have a human subject protections review board<br>Other, explain:
**Will the collaborating institution(s) be engaged in the research?**<br>(http://www.hhs.gov/ohrp/humansubjects/guidance/engage08.html)<br>No<br>Yes
**Will Virginia Tech's IRB review all human subject research activities involved with this project?**<br>No, provide the name of the primary institution:<br>Yes<br><br>*Note: primary institution = primary recipient of the grant or main coordinating center*

1.3 IS THIS RESEARCH FUNDED?

No, go to question 1.4
Yes, answer questions within table

IF YES

Provide the name of the sponsor [if NIH, specify department]: **NIH, NIDDK**

Is this project receiving federal funds?
No
Yes

If yes,

Does the grant application, OSP proposal, or "statement of work" related to this project include activities involving human subjects that are <u>not</u> covered within this IRB application?
No, all human subject activities are covered in this IRB application
Yes, however these activities will be covered in future VT IRB applications, these activities include:
Yes, however these activities have been covered in past VT IRB applications, the IRB number(s) are as follows:
Yes, however these activities have been or will be reviewed by another institution's IRB, the name of this institution is as follows: **For the subcontract to Brown University that involves conducting a pilot study, Brown University's IRB will review that protocol and related Human Subjects materials.**
Other, explain:

Is Virginia Tech the primary awardee or the coordinating center of this grant?
No, provide the name of the primary institution:
Yes

1.4 DOES THIS STUDY INVOLVE CONFIDENTIAL OR PROPRIETARY INFORMATION (OTHER THAN HUMAN SUBJECT CONFIDENTIAL INFORMATION), OR INFORMATION RESTRICTED FOR NATIONAL SECURITY OR OTHER REASONS BY A U.S. GOVERNMENT AGENCY?

For example – government / industry proprietary or confidential trade secret information

No
Yes, describe:

1.5 DOES THIS STUDY INVOLVE SHIPPING ANY TANGIBLE ITEM, BIOLOGICAL OR SELECT AGENT OUTSIDE THE U.S?

No
Yes

Section 2: Justification

2.1 DESCRIBE THE BACKGROUND, PURPOSE, AND ANTICIPATED FINDINGS OF THIS STUDY:

Pre-diabetes is present is more than half of adults aged 60-74 years. Resistance (strength) training appears to be another modality besides weight loss and physical activity that is effective for pre-diabetes prevention. Even though these health benefits can be accrued from a limited time investment, only 10-15% of people over 55 report performing any strengthening activities. Prior work suggests that resistance training (RT) can be effectively initiated in well-supervised settings, however there are very few theory-based studies showing effective maintenance of RT in minimally supervised settings. In the absence of such theoretically based efficacy studies on long-term maintenance, it is likely that although people such as those with pre-diabetes will initiate RT, a high percentage will soon discontinue RT. Therefore, we propose to demonstrate the efficacy of a social cognitive theory-based intervention for initiating and maintaining RT in older adults with pre-diabetes to improve glucose homeostasis. In this study, social cogntive theory with an emphasis on developing self-efficacy for RT and specific self-regulation strategies will form the foundation for one maintenance treatment with long-term though faded contact. A second maintenance treatment will follow the same contact plan as the first treatment but use only general content. A third treatment will follow present standard of care practices and use only general content and more limited contact. The design provides the ability to assess the long-term impacts of an intervention with theortically based components compared to a treatment of the same contact schedule and duration while both treatments also can be compared for public health importance to the present standard of care.

2.2 EXPLAIN WHAT THE RESEARCH TEAM PLANS TO DO WITH THE STUDY RESULTS:

For example - publish or use for dissertation

Results will be submitted for publication in a scientific journal.

Section 3: Recruitment

3.1 DESCRIBE THE SUBJECT POOL, INCLUDING INCLUSION AND EXCLUSION CRITERIA AND NUMBER OF SUBJECTS:

Examples of inclusion/exclusion criteria - gender, age, health status, ethnicity

The overall study will be preceded by formative work involving elicitation interviews with several to five men and women, ages 50-65, free of disease, who are consistent resistance trainees. A simple consent form will only be used for the inteviews that will be recorded with the audiotape erased after the interviewer checks notes for completeness. For the overall study, men and women will be eligible for the study if they are 50-69 years old, have a BMI between 25.0 to 39.9, are sedentary (i.e., less than 30 minutes of moderate physical activity, 5 days a week or less than 20 minutes vigorous physical activity, 3 days a week (ACSM, 2005), if they are not presently resistance training and have not done so in the past year, if they have no medical exclusions, and if they receive any required medical clearance (see below). We will recruit adults of all races and ethnic backgrounds; eligible individuals will be apparently healthy (no known presence of heart disease), with no symptoms of cardiovascular disease (chest discomfort, dizziness, shortness of breath, leg discomfort consistent with claudication) as defined by American Heart Association. Apparently healthy individuals, even those with >2 major cardiovascular risk factors such as impaired fasting glucose and obesity, may safely begin unsupervised low- and moderate-intensity exercise programs. The AHA/ACSM Health/Fitness Facility Preparticipation Screening Questionnaire will be used as part of the online eligibility screening process to confirm health status and symptoms suggestive of cardiovascular disease that would necessitate a pre-participation medically supervised graded exercise test and/or a higher level of supervision during exercise sessions Individuals who appear eligible following completion of the online screening will be required to obtain the written consent of their personal physician prior to enrollment using a standardized form, which also describes the nature of the study; this conservative approach will enable us to obtain medical clearance and contact information from participants' personal physicians as well as to inform personal physicians of their patient's participation in the research study. To participate, individuals must report being weight stable (+/-2 kg for the previous year), with a current body mass index in the overweight-to-obese range (BMI 25 – 39.9 kg/m2).   Eligible subjects (N=160-180) will be sedentary or minimally physically active, defined as no regular physical activity (<150 min/wk of moderate or <60 min/wk of vigorous PA) for at least one year prior to the study, and will not be presently resistance training or within the last year. To be eligible, participants must be willing and able to travel to a designated RT facility.

Medical Exclusions: Individuals will be excluded from participation if they are smokers, or if they have been diagnosed with cardiovascular disease (e.g., uncontrolled hypertension, coronary artery disease, previous MI), diabetes, pulmonary, liver or kidney disease, other life threatening illnesses or conditions (e.g., organ transplantation, HIV disease, cancer within the last 5 years). Individuals with conditions that restrict physical activity or the ability to resistance train such as orthopedic injuries or musculoskeletal disabilities will be excluded, as will those reporting a previously-diagnosed eating disorder. Individuals will also be excluded if they report absolute contraindications to RT, as described by the American Heart Association. Individuals will be excluded if they have had catacract surgery within the last two months, or if they have been diagnosed with retinophy. They will be excluded if are taking any medications known to influence energy metabolism, body weight or composition (e.g., thyroid replacement). Individuals taking commonly prescribed medications, such as those used to treat dyslipidemia and hormone replacement therapy, will be eligible for participation provided that they have been on a stable dose of the medication for an extended period of time (e.g., >1 year). A list of medication exclusions will be developed by the project Medical Director, Soheir Boshra, MD. Individuals with hypertension whose blood pressure is adequately controlled (i.e. <140/<90 mmHg) with antihypertensive medications will be permitted to participate, provided that their personal physician provides written permission for them to do so. Additional medical exclusion criteria used by Diabetes Prevention Program will be incorporated into our initial online self-screening process.

We plan to enroll a totoal of 160-180 individuals into this study; this will be divivided into four study cohorts.

3.2 WILL EXISTING RECORDS BE USED TO IDENTIFY AND CONTACT / RECRUIT SUBJECTS?

Examples of existing records - directories, class roster, university records, educational records

No, go to question 3.3
Yes, answer questions within table

IF YES
**Are these records private or public?**<br>Public<br>Private, describe the researcher's privilege to the records:
**Will student, faculty, and/or staff records or contact information be requested from the University?**<br>No<br>Yes, visit the following link for further information: http://www.policies.vt.edu/index.php (policy no. 2010)

3.3 DESCRIBE RECRUITMENT METHODS, INCLUDING HOW THE STUDY WILL BE ADVERTISED OR INTRODUCED TO SUBJECTS:

Recruitment will consist of the following strategies: 1) Hard copy and online stories and ads in local newspapers; contacts with area print and radio and TV reporters will be utilized to disseminate information about the project through different media outlets. 2) Church-bulletin inserts in local churches. If necessary, additional recruitment methods may include: 1) E-mail and print ads delivered directly to Carilion employees, via their Employee Wellness Division; 2) Advertisement during the annual Roanoke Valley League of Older Americans (LOA) community wellness screening conducted by the project Medical Director's clinic, the Carilion Center for Healthy Aging (>200 older adults screened per year); and 3) Print ads through the local LOA. During the recruitment process, interested people will be directed to a Web site for initial consent for screening and eligibility.

3.4 PROVIDE AN EXPLANATION FOR CHOOSING THIS POPULATION:

Note: the IRB must ensure that the risks and benefits of participating in a study are distributed equitably among the general population and that a specific population is not targeted because of ease of recruitment.

Pre-diabetes is highly prevalent among older overweight adults; data from NHANES III (projected for the year 2000) indicated that pre-diabetes occurs in ~53% of adults aged 60-74 yrs. Diabetes prevalence is highest among adults > 60 yrs as compared to other segments of the population; thus prevention efforts targeting this high-risk target population are clearly needed.

Section 4: Consent Process

For more information about consent process and consent forms visit the following link: http://www.irb.vt.edu/pages/consent.htm

If feasible, researchers are advised and may be required to obtain signed consent from each participant unless obtaining signatures leads to an increase of risk (e.g., the only record linking the subject and the research would be the consent document and the principal risk would be potential harm resulting in a breach of confidentiality). Signed consent is typically not required for low risk questionnaires (consent is implied) unless audio/video recording or an in-person interview is involved. If researchers will not be obtaining signed consent, participants must, in most cases, be supplied with consent information in a different format (e.g., in recruitment document, at the beginning of survey instrument, read to participant over the phone, information sheet physically or verbally provided to participant).

4.1 CHECK ALL OF THE FOLLOWING THAT APPLY TO THIS STUDY'S CONSENT PROCESS:

Verbal consent will be obtained from participants
Written/signed consent will be obtained from participants
Consent will be implied from the return of completed questionnaire. Note: The IRB recommends providing consent information in a recruitment document or at the beginning of the questionnaire (if the study only involves implied consent, skip to Section 5 below)
Other, describe: **As described in detail below, initial consent will be conducted online prior to completion of the online eligibility screening questions (see Figure in Supporting Documents). Thus, this phase of the consent**

process will utilize an implied consent process. Eligible individuals will then complete the consent process during an in-person visit, when written consent will be obtained. Because we are adding a survey about the maintenance follow-up phase that was not part of the original plans and consent, we will be using a separate online consent form for that survey.

4.2 PROVIDE A GENERAL DESCRIPTION OF THE PROCESS THE RESEARCH TEAM WILL USE TO OBTAIN AND MAINTAIN INFORMED CONSENT:

The consent process will consist of several steps (see Figure in Supporting Documents). Our recruitment informational materials will direct potential participants to a website, which will permit them to determine if they meet our eligibility requirements via an online self-screening process. Prior to accessing the online screening form, potential participants must complete an initial online Informed Consent process using an Information Sheet. After providing their consent to proceed, they will complete an online screening form which inquires about age, height and weight, cardiovascular disease symptoms, health and medical history, medication usage, and the AHA/ACSM Health/Fitness Facility Preparticipation Screening Questionnaire. Individuals who remain eligible according to this self-screening process will then be invited to proceed with enrollment by obtaining medical clearance from their personal physician. Written consent of the individual's personal physician will be obtained using a downloadable, HIPPA-compliant standardized medical clearance form, which will describe the nature of the study. This conservative approach will enable us to obtain medical clearance and contact information from participants' personal physicians as well as to inform personal physicians of their patient's participation in the research study. At this point in the online screening process, individuals will also download the Informed Consent Document (ICD) from the online screening site so that they are aware of all study requirements, risks and benefits of participation.

Interested individuals will send (via mail or fax) the completed, signed medical clearance form to the project director (PD) who will review and document clearance forms. Individuals will then be contacted via phone by either the PD or other research study staff person, to ask if the individual has any questions about the study or ICD contents; if they remain interested, the individual will be scheduled for the initial Screening and Assessment Clinic visit.

At the initial Screening and Assessment Clinic visit, individuals will be asked to return their signed ICD, and if they do not have this with them, they will be permitted to re-read/sign the ICD. They will again be asked if they have any questions regarding study participation at that time, before any testing is conducted. They will be provided with a copy of the signed ICD for their personal use. Asnoted, a separate online consent is being used for the follow-up survey.

4.3 WHO, FROM THE RESEARCH TEAM, WILL BE OVERSEEING THE PROCESS AND OBTAINING CONSENT FROM SUBJECTS?

The project director will be the primary individual responsible for obtaining the ICDs as well as overseeing the consent process.

4.4 WHERE WILL THE CONSENT PROCESS TAKE PLACE?

The consent process will take place first at the time of online screening for eligibility (this will be implicit initially, following review of the online Information Sheet and completion of the online eligibility screening questions), and then for eligible individuals, during the first in-person visit to the Assessment Clinic. The survery consent is done online.

4.5 DURING WHAT POINT IN THE STUDY PROCESS WILL CONSENTING OCCUR?

Note: unless waived by the IRB, participants must be consented before completing any study procedure, including screening questionnaires.

The consent process will be initiated before any information is obtained from participants, both online and in-person.

4.6 IF APPLICABLE, DESCRIBE HOW THE RESEARCHERS WILL GIVE SUBJECTS AMPLE TIME TO REVIEW THE CONSENT DOCUMENT BEFORE SIGNING:

Note: typically applicable for complex studies, studies involving more than one session, or studies involving more of a risk to subjects.

Individuals will have two opportunities to read the consent document (onlilne, and in-person at the first study visit), and two opportunites to ask questions about the study, participant expectations, and risks/ benefits of participation (phone call to schedule initial visit, and upon arrival to the testing site for the Assessment Clinic visit. The survey will be announced via an email to participants and participapnts will complete the online consent form before answering any survy questions.

Not applicable

Section 5: Procedures

5.1 PROVIDE A STEP-BY-STEP THOROUGH EXPLANATION OF ALL STUDY PROCEDURES EXPECTED FROM STUDY PARTICIPANTS, INCLUDING TIME COMMITMENT & LOCATION:

All study assessments will take place at the VT Riverside Research facility, located at 1703 South Jefferson Street, in Roanoke. The second floor of this building houses research space utilized by HNFE faculty.

Particants enrolled in this study will complete a 15-month Resistance Training (RT) intervention, described in brief as follows: All participants will first follow the same standard, supervised three-month initiation period with RT that previous research showed to be effective in this population. After the three-month initiation period, all people completing the Initial Phase will be randomly assigned to one of two maintenance conditions: 1. a long-term theory-based ASPIRE intervention, emphasizing self-regulation and other strategies to optimize training, with faded contact; 2. or, 2. a Standard intervention with more minimal contact. The RT sessions will occur twice per week, and will take 45 minutes or less to complete. RT sessions will take place at VT Riverside in the Initial Phase, all of which will be supervised by a research team member who is a certified Personal Trainer; RT sessions will take place at an RT facility of the participant's choice for subsequent study phases.

The primary outcome measures of this investigation are indices of pre-diabetes (glucose tolerance and fasting glucose concentration) and strength. Secondary measures include beta-cell responsiveness, insulin sensitivity, and disposition index as determined by the oral glucose and C-peptide minimal model; adherence; fat-free mass; other indicators of health and metabolic fitness, and Social Cognitive Theory (SCT) measures. Assessments will be done at baseline and repeated at months 3, 9, and 15 of the intervention period. Assessments will consist of the folllowing:

Primary Outcome Measures.
Muscular Strength. Strength on the chest press and leg press are primary outcome measures. We will assess a 3 RM, belived to be a good indicant of strength, while possibly safer than a 1 RM. Prior to baseline assessment, all participants will have an extended orientation to and practice with these movements. Following orientation, the procedures consist of a general warm-up, a specific warm-up on each machine, and then the performance of five controlled repetitions where the last repetition is judged as ‘hard’ on a standard perceived exertion scale (RPE). The participant will then rest for 10 minutes. The resistance then will be increased 10%-20% on the chest press and leg press beyond the resistance used for completing five repetitions. The participant will perform three controlled repetitions at an RPE at the last repetition of ‘very hard’ with an inability to perform a fourth repetition. If this level of perceived exertion is not reached, the participant will rest for four minutes, and the resistance will be increased 5% to 10% and the procedure repeated. The resistance used for the 3 RM in the chest press and for the leg press is the strength measure at all assessment points. In order to assure uniformity, strength testing will follow the same procedures at each assessment point. After baseline 3RM testing, a participant will be instructed on the use of the other machines. Through procedures similar to testing but using the repetition form and duration described for the actual training protocol, a resistance for each machine including the chest press and leg press will be ascertained that allows completion of six repetitions with an RPE of ‘hard’ for the last repetition. That will be the starting resistance for each machine in the Initiation phase. Estimated time of completion is 60 minutes at baseline, and ~30 minutes at each follow-up assessment.

Indices of Pre-diabetes. Consistent with American Diabetes Association criteria, pre-diabetes will be determined using fasting plasma glucose concentrations (FPG) and oral glucose tolerance tests (OGTT). At screening, pre-diabetes will be defined as FPG of 95-125 mg/dl (impaired fasting glucose; IFG) or a 2-h plasma glucose concentration of 140-199 mg/dl after a 75g oral glucose load (impaired glucose tolerance; IGT). Both pre-diabetes assessment criteria will be included, as many individuals with IGT may not exhibit high fasting plasma glucose concentrations; conversely, using only IGT may underestimate the proportion of the study population having pre-diabetes. An important finding of the DPP was a reduction in the incidence of IFG and IGT within one year of lifestyle intervention (41). For this reason, achievement of normal fasting plasma glucose and 2 hr plasma glucose concentration will be included as a primary outcome measure.

Secondary Outcome Measures:
Insulin Secretion and Action. Previous work has demonstrated improvements in insulin sensitivity (SI) with RT, and improvements in SI are associated with lower diabetes risk. Evidence of improvements in glucose/ insulin metabolism will be assessed by determination of insulin sensitivity, ß-cell responsivity, and disposition index, during an oral glucose tolerance test (OGTT), at each assessment point. Participants will undergo a 2-hour, 7-sample OGTT, during which plasma samples will be obtained at time 0 (administration of the 75g glucose load), 10, 20, 30, 60, 90, and 120 minutes for assessment of glucose, insulin and C-peptide concentrations. Samples will be analyzed for plasma glucose concentrations onsite using a YSI 2300 STAT Plus glucose analyzer; duplicate samples (20 uL sample, insulin; 100uL sample, C-peptide) will be treated with aprotinin (Sigma-Aldrich), frozen (-80ºC), and stored onsite for batch analysis of insulin and C-peptide concentrations using commercially available assay kits (Human Insulin ELISA; Human C-peptide RIA; [illegible]

5.2 DESCRIBE HOW DATA WILL BE COLLECTED AND RECORDED:

Data collected from participants includes self-reports ('paper and pen') on questionnaires, surveys, training logs, reports of food consumption via the Food Frequency Questionnaire, and ratings of self-efficacy and affect; laboratory biochemical assessments including fasting plasma glucose concentrations (FPG) and oral glucose tolerance test (OGTT) data; and strength assessed following standard procedures in our lab-gym. Other secondary measures include assessments of height and weight, body composition using DEXA, blood pressure, and steps per day using pedometers. All data collected will be recorded and maintained in hard copy form within each participants study file. Study files will be maintained in a locked file room within the VT Riverside facility, which is accessible only to study personnel. All data will only be identified with a subject study ID number. No names or other identifying information will be maintained in the study files.

Signed consent forms and participant contact information (names, phone nubmers, email addresses, mailing addresses, personal physician contact information, medical clearance forms) will be kept separate from the study files, in a locked filing cabinet within the PD's office at VT Riverside.

5.3 DOES THE PROJECT INVOLVE ONLINE RESEARCH ACTIVITES (INCLUDES ENROLLMENT, RECRUITMENT, SURVEYS)?

View the "Policy for Online Research Data Collection Activities Involving Human Subjects" at http://www.irb.vt.edu/documents/onlinepolicy.pdf

No, go to question 6.1
Yes, answer questions within table

IF YES

Identify the service / program that will be used:

www.survey.vt.edu, go to question 6.1
Blackboard, go to question 6.1
Center for Survey Research, go to question 6.1
Other

IF OTHER:

Name of service / program: **The online portions of this research project will be hosted by PC Resources, Inc. The domain name for this project is resist-diabetes.com. It will be determined based on a combination of the following: the preferences of the principal investigators and the availability of a domain name that is either exactly or as close to what is preferred by the researchers. A secure socket layer (SSL) certificate will be purchased and installed on the PC Resources Inc server so that all log in and data collection pages are properly encrypted during their transmission from the client computer to the server.**

Among the domains that are currently available, the following are possible:

resist-diabetes.com
train-for-health.com
no-to-diabetes.com

URL:

This service is…

Included on the list found at: http://www.irb.vt.edu/pages/validated.htm
Approved by VT IT Security
An external service with proper SSL or similar encryption (https://) on the login (if applicable) and all other data collection pages.
None of the above (note: only permissible if this is a collaborative project in which VT individuals are only responsible for data analysis, consulting, or recruitment)

Section 6: Risks and Benefits

6.1 WHAT ARE THE POTENTIAL RISKS (E.G., EMOTIONAL, PHYSICAL, SOCIAL, LEGAL, ECONOMIC, OR DIGNITY) TO STUDY PARTICIPANTS?

The project will use standard RT protocols with standard RT machines that follow ACSM's guidelines. Resistance training is specifically recommended for this age group and has been shown to be safe with low risk of injury. All participants will receive up to 24 personal training sessions with a personal training where proper technique will be emphasized. After this Initial Phase, all participants will continue to train. Some participants will have continued, though faded contact with a trainer, some will not. Unsupervised training can increase risks of injury if proper form and training algorithms are not followed. Participants will be asked to contact the principal investigators in the case of any injury or excessive joint or muscle soreness.
In addition to the possible risk of muscle soreness and excessive soreness in joints, other possible physical risks and adverse events that may be encountered with RT include acute increases in blood pressure and heart rate; however, these responses are very unlikely to occur with appropriate instruction and supervision. In individuals with retinopathy, vigorous RT may lead to vitreous hemorrhage and retinal detachment; in general, vigorous physical activity increases risk of sudden cardiac death and myocardial infarction in those with preexisting heart disease however absolute risk of these events is low (i.e. 1 event/60,000 participants hours). Prior RT studies in populations similar to that proposed in this application report no major injuries, other than transient musculoskeletal soreness (see grant references 46, 113, 114).

Features of the proposed RT intervention which will minimize possible risks include the screening procedures, supervised Initial Phase during which certified trainers will emphasize proper form and breathing techniques, the use of machines as opposed to free weights, and the use of moderate resistance, and gradual progressions of the training protocol. To enhance participant safety in later phases of the intervention, participants will be provided with emergency contact information for Dr. Boshra (the project Medical Director), an adverse event (AE) tracking log, recommendations for local RT facilities which have been evaluated by the PIs for the presence of certified staff, onsite emergency equipment (ie, AEDs) and safety procedures. In year 1, Dr. Boshra and her Clinical Nurse Specialist at the Center for Healthy Aging, Martha Anderson, will assist the PIs in developing study procedural manuals for clinical measurement procedures, in refining the AE notification and follow-up process and in participant recruitment efforts through the Center for Healthy Aging Clinic and annual community wellness screenings.

Presently, while there are different machines and implements that can be used to resistance train, there are no other treatments that can systematically increase strength, muscle mass, and consistently produce the numerous benefits of resistance training.

Additional possible risks related to Assessment Clinic procedures include radiation exposure from the DEXA scans, and risks associated with blood drawing during the OGTTs, such as bruising, infection at the catheter site, dizziness, and hypoglycemia. The amount of radiation received during each DEXA scan is comparable to 1/20th of a chest x-ray, and this amount of radiation exposure is not believed to greatly increase risk of developing cancerous tumors, however the exact increase in such risk is not known. Risks related to the OGTT procedures will be minimized by utilizing a trained, licensed nurse to insert catheters and obtain blood samples using aseptic techniques; participants will be monitored for dizziness and hypoglycemia during the OGTT by the research nurse, and orange juice and other snacks will be available in the event of a hypoglycemic episode.

6.2 EXPLAIN THE STUDY'S EFFORTS TO REDUCE POTENTIAL RISKS TO SUBJECTS:

Several processes will be in place to minimize potential risks during the investigation. First, physician medical clearnance will be required of all participants prior to enrollment. Second, the medical director will assist with the development of procedural manuals for clinical measurement procedures to enhance quality control and participtant safety. Third, certified personal trainers will be utilized to "train" study participants; all study staff members will also participate in CPR and AED certification programs through the VT Recreation Services Department. Finally, a Data and Safety Monitoring Board (DSMB) will be utilized. Unless there are adverse reactions to be reported monthly, Safety Reports will be forwarded to the DSMB at 6-month intervals at least 5 days prior to the DSMB meeting with the review committee. Information will be forwarded to NIH/NIDDK in the annual progress reports unless otherwise noted below. DSMB members and Safety Report format is listed in the attached grant application.

With regard to possible AEs: We anticipate adverse events will almost exclusively consist of mild muscle soreness plus a very low likelihood of other adverse events as delineated above. Based on ACSM (2005) data, with the population targeted by the proposed project, the likelihood of cardiac events or any other health risk as a result of the intervention is very minimal. However, participants will be instructed about possible AEs and be able to report these problems to the Medical Director via phone, via an email link on the intervention web site or by calling our project number, or informing their personal trainer. Participants will receive an AE tracking log which details possible symptoms, and will be instructed to record their symptoms, and to notify Dr. Boshra if any symptoms are of concern to them or if the symptoms last longer than would be expected. For emergencies, participants will be instructed to go directly to the emergency room, or to call 911. For “minor” problems, we will respond immediately and recommend the participant stop their program and consult a health care provider. The contacts will be secure, stored confidentially, monitored daily, and accessible only by program staff. Symptoms which occur will be discussed with Dr. Boshra, and if necessary, she will instruct us to withdraw the treatment, recommend the participant stop the program, and consult with their health care provider. Personal trainers through their training and ongoing supervision, will be educated about maintaining participant confidentiality regarding all participant information, especially medical, health, and demographic information, or changes in medical history that may become apparent during the course of the intervention. The personal trainers will be asked to include any signs of such in their notes and records of training sessions; any signs will be reported immediately to Dr Boshra and the study PIs. For any problem, we will initiate a follow-up contact to assure the participant was able to follow-up with a health care provider, and we will require medical clearance for such participants to proceed in the program. We will keep records of these reports so that we will be able to assess if a specific participant repetitively reports problems. If there are three such instances within a three-month period, we will ask the participant to discontinue participation.

All these adverse events will be reported within 5-6 days to our IRB and every six months to the NIH/NIDDK, should they occur. For any new problem (AE) that occurs that was not completely indicated on a prior consent form, we will notify all participants via email of its occurrence plus stesp to take if they experience the same symptoms.

6.3 WHAT ARE THE DIRECT OR INDIRECT ANTICIPATED BENEFITS TO STUDY PARTICIPANTS AND/OR SOCIETY?

Participant Benefits. All participants can obtain at least 24 personal training sessions with a personal trainer. The type of protocol that will be used has been shown to produce favorable changes in mechanism associated with diabetes, coronary heart disease, cancers, and disabilities. Resistance training in this form is recommended for this age group. Because of the inclusion, exclusion criteria and screening, there are few risks for performing the resistance training protocols. Participants will be instructed, practice, and learn a way of resistance training that can be performed for the rest of their functional life.

Importance of Knowledge to be Gained. The study can provide important data about a theoretical approach to helping older adults maintain resistance, the theoretical mechanisms that are involved, and the ability to prevent Type 2 diabetes. Resistance training is recommended for older adults, but only a small percent of this age group is involved in any strengthening exercise. Given the design, the study will be able to show if a theory-based approach with faded but extended contact provides better health-related outcomes than a more standard intervention with faded contact. The study also will be able to assess the public health issue assessing the efficacy of either of these interventions.

Section 7: Full Board Assessment

7.1 DOES THE RESEARCH INVOLVE MICROWAVES/X-RAYS, OR GENERAL ANESTHESIA OR SEDATION?

No
Yes

7.2 DO RESEARCH ACTIVITIES INVOLVE PRISONERS, PREGNANT WOMEN, FETUSES, HUMAN IN VITRO FERTILIZATION, OR MENTALLY DISABLED PERSONS?

No, go to question 7.3
Yes, answer questions within table

IF YES
**This research involves:**<br>Prisoners<br>Pregnant women Fetuses Human in vitro fertilization<br>Mentally disabled persons

7.3 DOES THIS STUDY INVOLVE MORE THAN MINIMAL RISK TO STUDY PARTICIPANTS?

Minimal risk means that the probability and magnitude of harm or discomfort anticipated in the research are not greater in and of themselves than those ordinarily encountered in daily activities or during the performance of routine physical or psychological examinations or tests. Examples of research involving greater than minimal risk include collecting data about abuse or illegal activities. Note: if the project qualifies for Exempt review (http://www.irb.vt.edu/pages/categories.htm), it will not need to go to the Full Board.

No
Yes

IF YOU ANSWERED "YES" TO ***ANY ONE*** OF THE ABOVE QUESTIONS, 7.1, 7.2, OR 7.3, THE BOARD MAY REVIEW THE PROJECT'S APPLICATION MATERIALS AT ITS MONTHLY MEETING. VIEW THE FOLLOWING LINK FOR DEADLINES AND ADDITIONAL INFORMATION: http://www.irb.vt.edu/pages/deadlines.htm

Section 8: Confidentiality / Anonymity

For more information about confidentiality and anonymity visit the following link: http://www.irb.vt.edu/pages/confidentiality.htm

8.1 WILL PERSONALLY IDENTIFYING STUDY RESULTS OR DATA BE RELEASED TO ANYONE OUTSIDE OF THE RESEARCH TEAM?

For example – to the funding agency or outside data analyst, or participants identified in publications with individual consent

No
Yes, to whom will identifying data be released?

8.2 WILL ANY STUDY FILES CONTAIN PARTICIPANT IDENTIFYING INFORMATION (E.G., NAME, CONTACT INFORMATION, VIDEO/AUDIO RECORDINGS)?

Note: if collecting signatures on a consent form, select "Yes."

No, go to question 8.3
Yes, answer questions within table

IF YES
**Describe if/how the study will utilize study codes: Each participant will be assigned a study ID number, and this number will be used to code all data pertaining to that individual. A list of study ID numbers with participants names (key) will be maintained by the project director, in their office at the VT Riverside facility. The study PIs will also have access to this key, and if needed for certain circumstances where medical follow-up is needed, also the project Medical Director. Any documents with identifying information, such as signed consent forms and medical clearance forms, will be maintained in the project director's office in a location separate from the study data files, and separate from the key. The data files (containing only the study ID numbers, and no identifying information) will be kept in a locked data storage room in the VT Riverside facility, in a locked filing cabinet. This room and filing cabinet will be accessible to all study personnel.**
**If applicable, where will the key** [i.e., linked code and identifying information document (for instance, John Doe = study ID 001)] **be stored and who will have access? The key to the codes will be stored in the office of the project director, which will be accessible to the study PIs. Only the project director and study PIs will have access to this information, unless there is an adverse event or other medical siutation, which involves the study Medical Director, who will also have access to the key under these circumstances.**<br><br>*Note: the key should be stored separately from subjects' completed data documents and accessibility should be limited.*
The IRB strongly suggests and may require that all data documents (e.g., questionnaire responses, interview responses, etc.) do not include or request identifying information (e.g., name, contact information, etc.) from participants. If you need to link subjects' identifying information to subjects' data documents, use a study ID/code on all data documents.

8.3 WHERE WILL DATA BE STORED?

Examples of data - questionnaire, interview responses, downloaded online survey data, observation recordings, biological samples

All data in "hard copy" form will be stored at the VT Riverside Research facility in a locked file rooom, accessible to only study personel. All data collected online will be stored in a password-protected database on the PC Resources Inc. server. The only individuals who have password access to the data will be the PC Resources staff assigned to the project. PI's will have indirect access to the data via a restricted segment of the site specifically designed to display reports of the results. Only individuals designated by the PIs will be granted access to the reporting site.

8.4 WHO WILL HAVE ACCESS TO STUDY DATA?

Study personel only (PIs, Medical Director, Project Director, study staff).

8.5 DESCRIBE THE PLANS FOR RETAINING OR DESTROYING THE STUDY DATA

This investigation will take 5 years to complete; all data will be maintained in a secure location for at least ten years beyond the study completion date. After that time, data may be be destroyed.

8.6 DOES THIS STUDY REQUEST INFORMATION FROM PARTICIPANTS REGARDING ILLEGAL BEHAVIOR?

No, go to question 9.1

Yes, answer questions within table

IF YES
**Does the study plan to obtain a Certificate of Confidentiality?**<br>No<br>Yes (Note: participants must be fully informed of the conditions of the Certificate of Confidentiality within the consent process and form)<br><br>*For more information about Certificates of Confidentiality, visit the following link: http://www.irb.vt.edu/pages/coc.htm*

Section 9: Compensation

For more information about compensating subjects, visit the following link: http://www.irb.vt.edu/pages/compensation.htm

9.1 WILL SUBJECTS BE COMPENSATED FOR THEIR PARTICIPATION?

No, go to question 10.1
Yes, answer questions within table

IF YES
**What is the amount of compensation? Participants will receive a minimal payment of $15-30 for assessments in the form of gift cards.**
**Will compensation be prorated?**<br>Yes, please describe:<br>No, explain why and clarify whether subjects will receive full compensation if they withdraw from the study? **Participants will receive a minimal payment of $15-$30 for assessments in the form of gift cards.**<br><br>*Unless justified by the researcher, compensation should be prorated based on duration of study participation. Payment must not be contingent upon completion of study procedures. In other words, even if the subject decides to withdraw from the study, he/she should be compensated, at least partially, based on what study procedures he/she has completed.*

Section 10: Audio / Video Recording

For more information about audio/video recording participants, visit the following link: http://www.irb.vt.edu/pages/recordings.htm

10.1 WILL YOUR STUDY INVOLVE VIDEO AND/OR AUDIO RECORDING?

No, go to question 11.1
Yes, answer questions within table

IF YES

**This project involves:**<br>Audio recordings only<br>Video recordings only<br>Both video and audio recordings
**Provide compelling justification for the use of audio/video recording:**
**How will data within the recordings be retrieved / transcribed?**
**How and where will recordings (e.g., tapes, digital data, data backups) be stored to ensure security?**
**Who will have access to the recordings?**
**Who will transcribe the recordings?**
**When will the recordings be erased / destroyed?**

Section 11: Research Involving Students

11.1 DOES THIS PROJECT INCLUDE STUDENTS AS PARTICIPANTS?

No, go to question 12.1
Yes, answer questions within table

IF YES
**Does this study involve conducting research with students of the researcher?**<br>No<br>Yes, describe safeguards the study will implement to protect against coercion or undue influence for participation:<br><br>*Note: if it is feasible to use students from a class of students not under the instruction of the researcher, the IRB recommends and may require doing so.*
**Will the study need to access student records (e.g., SAT, GPA, or GRE scores)?**<br>No<br>Yes

11.2 DOES THIS PROJECT INCLUDE ELEMENTARY, JUNIOR, OR HIGH SCHOOL STUDENTS?

No, go to question 11.3
Yes, answer questions within table

IF YES

Will study procedures be completed during school hours?

No
Yes

If yes,

Students not included in the study may view other students' involvement with the research during school time as unfair. Address this issue and how the study will reduce this outcome:

Missing out on regular class time or seeing other students participate may influence a student's decision to participate. Address how the study will reduce this outcome:

Is the school's approval letter(s) attached to this submission?

Yes
No, project involves Montgomery County Public Schools (MCPS)
No, explain why:

You will need to obtain school approval (if involving MCPS, click here: http://www.irb.vt.edu/pages/mcps.htm). Approval is typically granted by the superintendent, principal, and classroom teacher (in that order). Approval by an individual teacher is insufficient. School approval, in the form of a letter or a memorandum should accompany the approval request to the IRB.

11.3 DOES THIS PROJECT INCLUDE COLLEGE STUDENTS?

No, go to question 12.1
Yes, answer questions within table

IF YES

Some college students might be minors. Indicate whether these minors will be included in the research or actively excluded:

Included
Actively excluded, describe how the study will ensure that minors will not be included:

Will extra credit be offered to subjects?

No
Yes

If yes,

What will be offered to subjects as an equal alternative to receiving extra credit without participating in this study?

Include a description of the extra credit (e.g., amount) to be provided within question 9.1 ("IF YES" table)

Section 12: Research Involving Minors

12.1 DOES THIS PROJECT INVOLVE MINORS (UNDER THE AGE OF 18 IN VIRGINIA)?

Note: age constituting a minor may differ in other States.

No, go to question 13.1
Yes, answer questions within table

IF YES
**Does the project reasonably pose a risk of reports of current threats of abuse and/or suicide?**<br>No<br>Yes, thoroughly explain how the study will react to such reports:<br><br>*Note: subjects and parents must be fully informed of the fact that researchers must report threats of suicide or suspected/reported abuse to the appropriate authorities within the Confidentiality section of the Consent, Assent, and/or Permission documents.*
**Are you requesting a waiver of parental permission (i.e., parent uninformed of child's involvement)?**<br>No, **both** parents/guardians will provide their permission, if possible.<br>No, **only one** parent/guardian will provide permission.<br>Yes, describe below how your research meets **all** of the following criteria (A-D):<br>Criteria A - The research involves no more than minimal risk to the subjects:<br>Criteria B - The waiver will not adversely affect the rights and welfare of the subjects:<br>Criteria C - The research could not practicably be carried out without the waiver:<br>Criteria D - (Optional) Parents will be provided with additional pertinent information after participation:
**Is it possible that minor research participants will reach the legal age of consent (18 in Virginia) while enrolled in this study?**<br>No<br>Yes, will the investigators seek and obtain the legally effective informed consent (in place of the minors' previously provided assent and parents' permission) for the now-adult subjects for any ongoing interactions with the subjects, or analysis of subjects' data? If yes, explain how:<br><br>*For more information about minors reaching legal age during enrollment, visit the following link: http://www.irb.vt.edu/pages/assent.htm*
*The procedure for obtaining assent from minors and permission from the minor's guardian(s) must be described in **Section 4** (Consent Process) of this form.*

Section 13: Research Involving Deception

For more information about involving deception in research and for assistance with developing your debriefing form, visit our website at http://www.irb.vt.edu/pages/deception.htm

13.1 DOES THIS PROJECT INVOLVE DECEPTION?

No, go to question 14.1
Yes, answer questions within table

IF YES
**Describe the deception:**

**Why is the use of deception necessary for this project?**
**Describe the debriefing process:**
**Provide an explanation of how the study meets <u>all</u> the following criteria (A-D) for an alteration of consent:**<br>Criteria A - The research involves no more than minimal risk to the subjects:<br>Criteria B - The alteration will not adversely affect the rights and welfare of the subjects:<br>Criteria C - The research could not practicably be carried out without the alteration:<br>Criteria D - (Optional) Subjects will be provided with additional pertinent information after participation (i.e., debriefing for studies involving deception):<br><br>*By nature, studies involving deception cannot provide subjects with a complete description of the study during the consent process; therefore, the IRB must allow (by granting an alteration of consent) a consent process which does not include, or which alters, some or all of the elements of informed consent.*
The IRB requests that the researcher use the title "Information Sheet" instead of "Consent Form" on the document used to obtain subjects' signatures to participate in the research. This will adequately reflect the fact that the subject cannot fully consent to the research without the researcher fully disclosing the true intent of the research.

Section 14: Research Involving Existing Data

14.1 WILL THIS PROJECT INVOLVE THE COLLECTION OR STUDY/ANALYSIS OF EXISTING DATA DOCUMENTS, RECORDS, PATHOLOGICAL SPECIMENS, OR DIAGNOSTIC SPECIMENS?

Please note: it is not considered existing data if a researcher transfers to Virginia Tech from another institution and will be conducting data analysis of an on-going study.

No, you are finished with the application
Yes, answer questions within table

IF YES
**From where does the existing data originate?**
**Provide a detailed description of the existing data that will be collected or studied/analyzed:**
**Is the source of the data public?**<br>No, continue with the next question<br>Yes, you are finished with this application

Will any individual associated with this project (internal or external) have access to or be provided with existing data containing information which would enable the identification of subjects:

- **Directly** (e.g., by name, phone number, address, email address, social security number, student ID number), or
- **Indirectly through study codes** even if the researcher or research team does not have access to the master list linking study codes to identifiable information such as name, student ID number, etc

or

- **Indirectly through the use of information that could reasonably be used in combination to identify an individual** (e.g., demographics)

No, collected/analyzed data will be completely de-identified
Yes,

If yes,

Research will not qualify for exempt review; therefore, if feasible, written consent must be obtained from individuals whose data will be collected / analyzed, unless this requirement is waived by the IRB.

Will written/signed or verbal consent be obtained from participants prior to the analysis of collected data?

This research protocol represents a contract between all research personnel associated with the project, the University, and federal government; therefore, must be followed accordingly and kept current.

Proposed modifications must be approved by the IRB prior to implementation except where necessary to eliminate apparent immediate hazards to the human subjects.

Do not begin human subjects activities until you receive an IRB approval letter via email.

It is the Principal Investigator's responsibility to ensure all members of the research team who collect or handle human subjects data have completed human subjects protection training prior to handling or collecting the data.

----------END----------
